# Response to the Letter: Analytic approaches for prognostic studies of persistent pain following knee arthroplasty

**DOI:** 10.2340/17453674.2026.46806

**Published:** 2026-09-07

**Authors:** Anni Rajamäki, Aleksi Reito, Mari Karsikas, Mika Niemeläinen, Antti Eskelinen

**Affiliations:** Coxa Hospital for Joint Replacement and Faculty of Medicine and Health Technology, Tampere University, Tampere, Finland

*Sir,—*We thank Dr Riddle and Dr Dumenci for their insightful comments to our article “Predicting persistent pain after total knee arthroplasty using different machine learning algorithms” [1,2]. They highlighted a few important points from the methodology and proposed an alternative statistical approach to predict the outcomes after total knee arthroplasty (TKA).

The first concern was raised regarding the fact that all outcome variables were derived from the Oxford Knee Score (OKS) and were dichotomized. We agree that these outcomes are related and that dichotomization results in some loss of information. However, they were not intended to represent statistically independent outcomes, as our main interest was to predict postoperative pain, while the prediction of the total OKS and an improvement in OKS below the MCID threshold were examined as additional outcomes. We defined the non-responder group as an improvement in OKS of less than 9 points, not 8 points [3]. The continuous outcome was dichotomized to allow its use as the target variable in the binary XGBoost classification model.

We would also like to clarify the issue of missing outcome data. The percentages cited in the letter (52–69%) reflect the proportion of the original registry population not included in the analyses because the required outcome data was unavailable, not the proportion of patients with missing outcome data among those included in the analyses. This limitation was explicitly acknowledged in our Discussion, where we noted that more than 12,000 patients without available OKS information were excluded and that socioeconomic and other patient characteristics may influence PROM response rates [2].

The authors proposed latent class growth modelling as a potentially superior prognostic approach for estimating the probability of a poor pain outcome in patients undergoing knee arthroplasty [4,5]. This approach may help avoid dichotomization and reliance on a strict MCID threshold. These methods could help characterize the patient population that does not improve after surgery. However, the main aim of our study was to build a prediction model to identify, using preoperative information only, patients at risk of residual pain or poor function. The latent class growth modelling approach relies on repeated postoperative outcome measurements to identify latent groups based on longitudinal outcome trajectories [5]. This can be regarded as a valuable complementary approach in this field for identifying the non-responder patient groups.

However, a poor AUC does not necessarily indicate a need to improve the machine-learning algorithms themselves. We conducted the analyses with 3 different algorithms and increasing methodological complexity did not improve predictive performance in our dataset. If the preoperative variables do not contain sufficient information to explain residual pain and poor function, more sophisticated statistical methodology is unlikely to substantially improve prediction. Persistent pain and patient-reported functional outcomes after TKA are complex and likely influenced by factors that are not captured by routinely collected preoperative clinical data. We thank the authors for raising these important methodological considerations and for introducing an alternative approach to predicting postoperative outcomes.

## References

[1] Riddle D L, Dumenci L. Letter to the Editor: Analytic approaches for prognostic studies of persistent pain following knee arthroplasty. Acta Orthop 2026; 97: 629-30. doi: 10.2340/17453674.2026.4669842703636

[2] Rajamäki A, Reito A, Karsikas M, Niemeläinen M, Eskelinen A. Predicting persistent pain after total knee arthroplasty using different machine learning algorithms. Acta Orthop 2026; 97: 423-9. doi:10.2340/17453674.2026.45965. 42329268 PMC13285364

[3] Beard D J, Harris K, Dawson J, Doll H, Murray D W, Carr A J, et al. Meaningful changes for the Oxford hip and knee scores after joint replacement surgery. J Clin Epidemiol 2015; 68(1): 73-9. doi:10.1016/j.jclinepi.2014.08.009. 25441700 PMC4270450

[4] Riddle D L, Dumenci L. Using two predictive models to capture two types of poor outcomes in knee arthroplasty: a multisite longitudinal cohort study. Arthritis Rheumatol 2024; 76(7): 1036-46. doi:10.1002/art.42819. 38327016 PMC11213671

[5] Dumenci L, Perera R A, Keefe F J, Ang D C, Slover J, Jensen M P, et al. Model-based pain and function outcome trajectory types for patients undergoing knee arthroplasty: a secondary analysis from a randomized clinical trial. Osteoarthritis Cartilage 2019; 27(6): 878–84. doi:10.1016/j.joca.2019.01.004. 30660721 PMC6536318

